# Application of microscopic smartphone attachment for remote preoperative lab testing

**DOI:** 10.3389/fdgth.2024.1461559

**Published:** 2024-11-28

**Authors:** Kefan Song, Alexander T. Adams

**Affiliations:** ^1^ Wallace H. Coulter Department of Biomedical Engineering, Georgia Institute of Technology, Atlanta, GA, United States; ^2^ Institute for Robotics and Intelligent Machines, Georgia Institute of Technology, Atlanta, GA, United States; ^3^ School of Interactive Computing, Georgia Institute of Technology, Atlanta, GA, United States

**Keywords:** preoperative testing, point-of-care, microscope, blood analysis, healthcare, pathology

## Abstract

**Introduction:**

Current preoperative exam guidelines utilize extensive lab tests, including blood tests and urine analysis, which are crucial for assessing surgical readiness. However, logistical challenges, especially for patients traveling long distances for high-quality medical care, create significant delays and burdens. This study aims to address these challenges by applying a previously developed point-of-care (POC) device system to perform accurate and rapid lab tests. This device is designed to assist both healthcare providers in resource-limited settings and patients by offering a low-cost, portable diagnostic tool that enables both in-clinic and at-home testing.

**Methods:**

The system was tested for adaptability and compatibility by transitioning from its original Android platform to an iOS platform. A custom application was developed to maintain the system’s capabilities of capturing optimal cell images across different mobile platforms. The system’s cell counting algorithm was tailored to process the captured images, featuring a streamlined workflow that includes image processing and automated cell detection using a Hough circle algorithm.

**Results:**

The new system provided good-quality raw images with 26.3 px/μm pixel resolution and 2.19 μm spatial resolution, facilitating effective cell recognition and counting. The cell counting algorithm demonstrated high precision (0.8663) and high recall (0.9312), with a correlation ($R^{2}=0.89535$) between algorithm-generated counts and actual counts.

**Discussion:**

This study highlights the potential of the POC device to streamline preoperative testing, making it more accessible and efficient, particularly for patients in rural areas or those needing to travel for medical care. Future enhancements, including wider field-of-view, adjustable magnification, more advanced and integrated algorithms as well as integration with a microfluidic channel for direct sample analysis, are proposed to expand the device’s functionality. The device’s portability, ease of use, and rapid processing time position it as a promising alternative to traditional lab tests, ultimately aiming to improve patient care and surgical outcomes.

## 1 Introduction

Clinical lab tests are part of the standardized preoperative exam guidelines observed in healthcare systems in the world. When testing is required preoperatively, blood tests are almost always performed since they are able to help assess a patient’s general health condition, evaluate organ function, identify bleeding disorders, as well as determine infection or inflammatory conditions. Urine sample analysis is also commonly ordered, especially for patients with urinary tract symptoms or undergoing urological procedures (1). A lot of information could be extracted from these tests, but most of this information involves the presence or concentration of certain cells or chemicals, such as full blood count, creatinine concentration, and hematuria (2).

Research studies have indicated that many routine preoperative tests are excessive and their results would not alter the surgical outcome (3, 4). However, current clinical guidelines still suggest the inclusion of such tests based on the historical findings and patient conditions (5). In modern practice, risk stratification algorithms are often used to determine which preoperative tests are required, based on a patient’s medical history, comorbidities, and the complexity of the procedure (6). While these algorithms help optimize patient care, they can also lead to a large number of tests, especially for high-risk patients. Performing this extensive testing often requires significant time and resources, contributing to delays in the preoperative process. The debate over whether to cut certain tests is ongoing, but a more practical solution would be to make these tests cheaper and easier to perform so that they don’t take up much time and resources. This approach addresses the financial and logistical barriers without compromising patient safety (7).

Many individuals seeking high-quality medical care travel significant distances to reach advanced medical facilities, far from their residences. They also face excess travel frequency for unnecessary preoperative testings, no matter how near or far they live from hospitals. Even in a well-developed country like the United States, this phenomenon is still notably prevalent, where more than 50% of patients received unindicated preoperative tests (8), and about a quarter of patients traveled more than 100 miles to receive congenital heart surgery (9). This distance poses substantial challenges, including transportation difficulties and the timely collection and examination of samples. Scheduling preoperative exams becomes particularly tricky because these tests are usually conducted shortly before the surgery. Backlogs at testing facilities and delays in obtaining test results have been shown to contribute to the postponement of elective surgeries (10). Additionally, involving a third-party medical facility nearby for preoperative testing only is not always feasible due to logistical issues and the need for careful appointment planning. In rural settings, the infrastructure might not support the timely transportation of samples to distant labs, potentially delaying diagnosis and treatment. This problem is compounded by the fact that many rural areas have limited access to specialized medical equipment and trained personnel, further hindering the efficiency of preoperative testing.

Moreover, the current lab test procedures require sending samples to specific labs where complicated processing steps are involved, resulting in delays in obtaining results. It also raises the issue of the availability of such labs, especially in underdeveloped areas. In most countries around the world, there is a significant shortage of both pathological lab equipment as well as personnel, which exacerbates the problem. This shortage means that many patients in remote or under-served regions may not have timely access to necessary preoperative tests, leading to delays in surgical procedures and potentially compromising patient outcomes. Additionally, the overburdened existing labs often struggle to keep up with the demand, leading to further delays and a backlog of tests (11). This situation underscores the urgent need for more efficient and accessible diagnostic solutions that can deliver accurate results quickly and reliably, without being dependent on centralized lab facilities. To address these challenges, there is a growing need for diagnostic solutions that do not rely on fully equipped laboratories or specialized personnel. Instead, these solutions aim to bring testing directly to the point of care, allowing for faster and more convenient testing without the need for large-scale infrastructure.

On the other hand, point-of-care (POC) devices have gained popularity and might offer a viable alternative to traditional clinical lab testing, as they can potentially streamline the testing process, providing results more quickly and efficiently. POC devices are designed to perform a variety of tests at or near the site of patient care, thereby reducing the need for sample transport and expediting the diagnostic process (7). The development of POC devices has also benefited from the fast advancement and widespread adoption of smartphone technologies, as the high data processing capabilities and powerful sensors of smartphones can be utilized for POC devices, thus eliminating hurdles in both cost and access to high-end technology (12). This is especially important in rural areas because even though their access to healthcare facilities and laboratories is the most limited (11), their ownership rate of smartphones is comparable to that of urban areas, at about 80% (13).

The advancement of POC technology has the potential to revolutionize preoperative testing. These devices are compact, easy to use, and can deliver accurate results rapidly, making them ideal for use in both rural and urban settings. The convenience and speed offered by POC testing can also facilitate more effective preoperative assessments, reducing the time patients spend waiting for test results and enabling quicker surgical interventions. Furthermore, POC devices integrated into smartphones can be designed to be user-friendly and mostly automatic, allowing patients with minimal training to perform complex diagnostic tests easily and accurately from their homes (14).

The need for smartphone-based POC devices extends beyond just convenience. The ability of the smartphone to provide and transmit timely data and rapid analysis of the collected sample allows for the full utilization of the professional pathologist network worldwide, so experts around the world could help guide sample collection procedures or review analysis results to eliminate patient constraints of local resource availability and accessibility (15).

**Figure 1:**
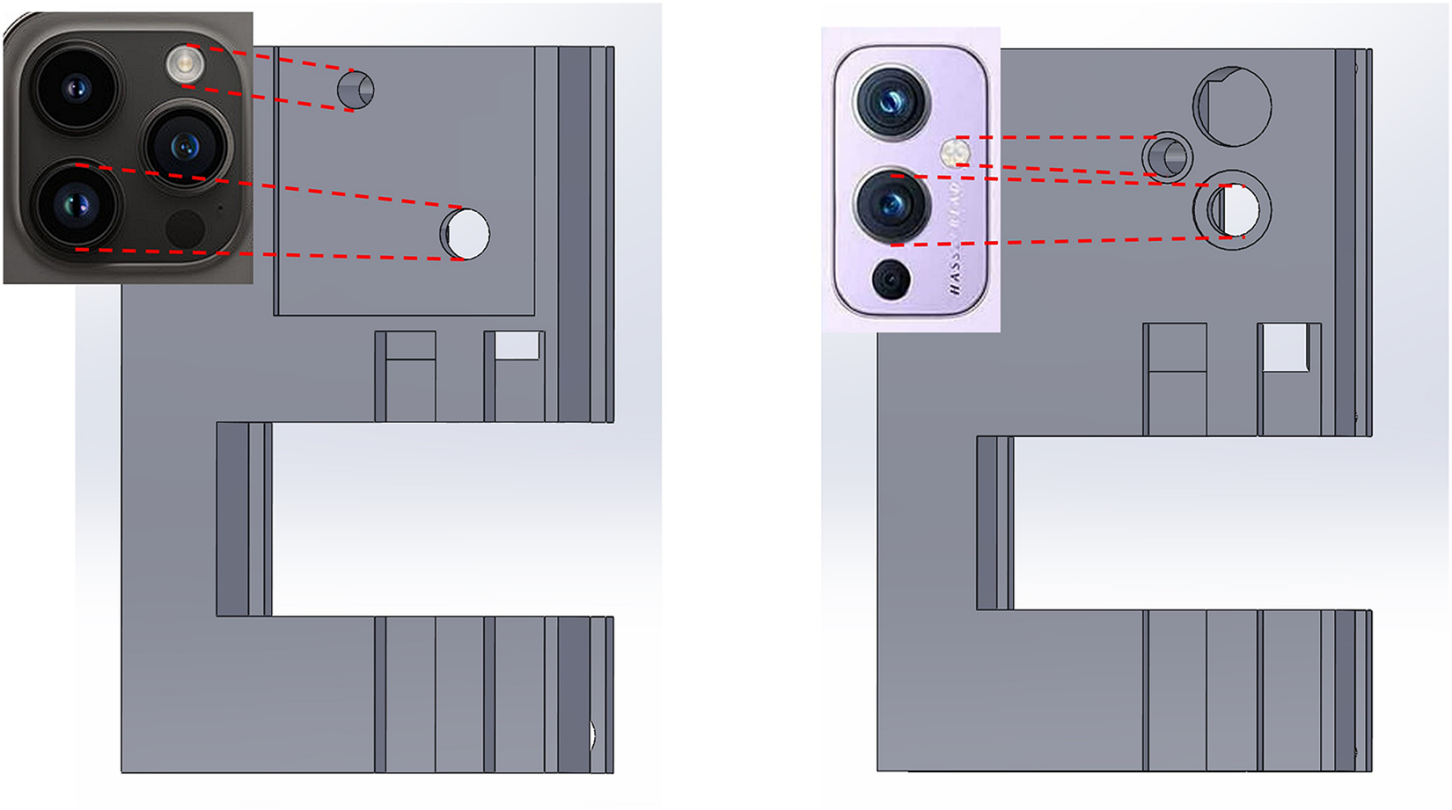
Left: Prototype model fitted to camera dimensions of an iPhone 14 Pro Max; Right: Prototype model fitted to camera dimension of an OnePlus 9.

Thus, there is a pressing need for a POC device that can perform lab tests accurately with a uniform testing scale, without being cost-prohibitive or logistically challenging. In our recently submitted work, we developed a microscope attachment for smartphones called μ-phone that aims to tackle this issue (16). The μ-phone utilizes inexpensive, accessible optical components packaged as a smartphone attachment, enabling microscopic image capture and analysis at a cellular level. Its compact form factor and ease of production make it a cost-effective solution for conducting blood tests in resource-limited settings. The initial prototype of the μ-phone was designed to work with an Android smartphone (OnePlus 9), but in this study, we demonstrate its versatility by adapting the system to an iOS device, requiring only adjustments to fit the size and location of the camera and flashlight on the desired smartphone model (Figure 1). While the hardware for this device was previously validated, this paper presents the novel integration of a custom app and a cell counting algorithm, creating a fully functional system for preoperative lab testing with high potential for deployment. Unlike traditional clinical microscopes, this device integrates both the hardware (the microscope) and the software (the cell counting algorithm) into a single system that can be used by non-specialists in a variety of settings. This eliminates the need for both a full laboratory and highly trained personnel for microscope-related tests, making preoperative testing more accessible to patients in underserved areas. In this paper, we discuss the application of this complete system, focusing on its ability to perform automated blood cell counting and its potential to adapt to major phone models. We also aim to highlight its capacity to perform other diagnostic tests, such as the identification and counting of specific cell types. The integration of the custom app with the hardware allows for real-time data collection and processing, enabling faster and more accurate preoperative assessments in resource-limited settings. This device is designed for use by both care providers and patients. For care providers, it reduces the need for extensive clinical laboratory setups, lowering financial and logistical barriers, especially in remote or resource-limited environments. For patients, it offers the potential for more frequent and convenient health monitoring, eliminating the need for travel to distant healthcare facilities for preoperative tests. By empowering patients with a portable, easy-to-use diagnostic tool, the system enhances patient autonomy and reduces delays in care. This innovation could streamline the preoperative process, allowing for more efficient scheduling and reducing the overall time and resources spent on preoperative assessments.

## 2 Materials and methods

Building on the system’s potential for universal adaptability, we tested the μ-phone for compatibility across multiple smartphone platforms, transitioning it from its original Android implementation to an iOS device. This adaptation was part of a broader effort to ensure that the device could be easily applied to different phone models while maintaining consistent and accurate cell counting performance. The goal was to verify that the hardware could be adapted to new smartphones without significant modification, confirming the system’s versatility in resource-limited settings. As part of this proof-of-concept demonstration, we used the iPhone 14 Pro Max as a representative example.

**Figure 2:**
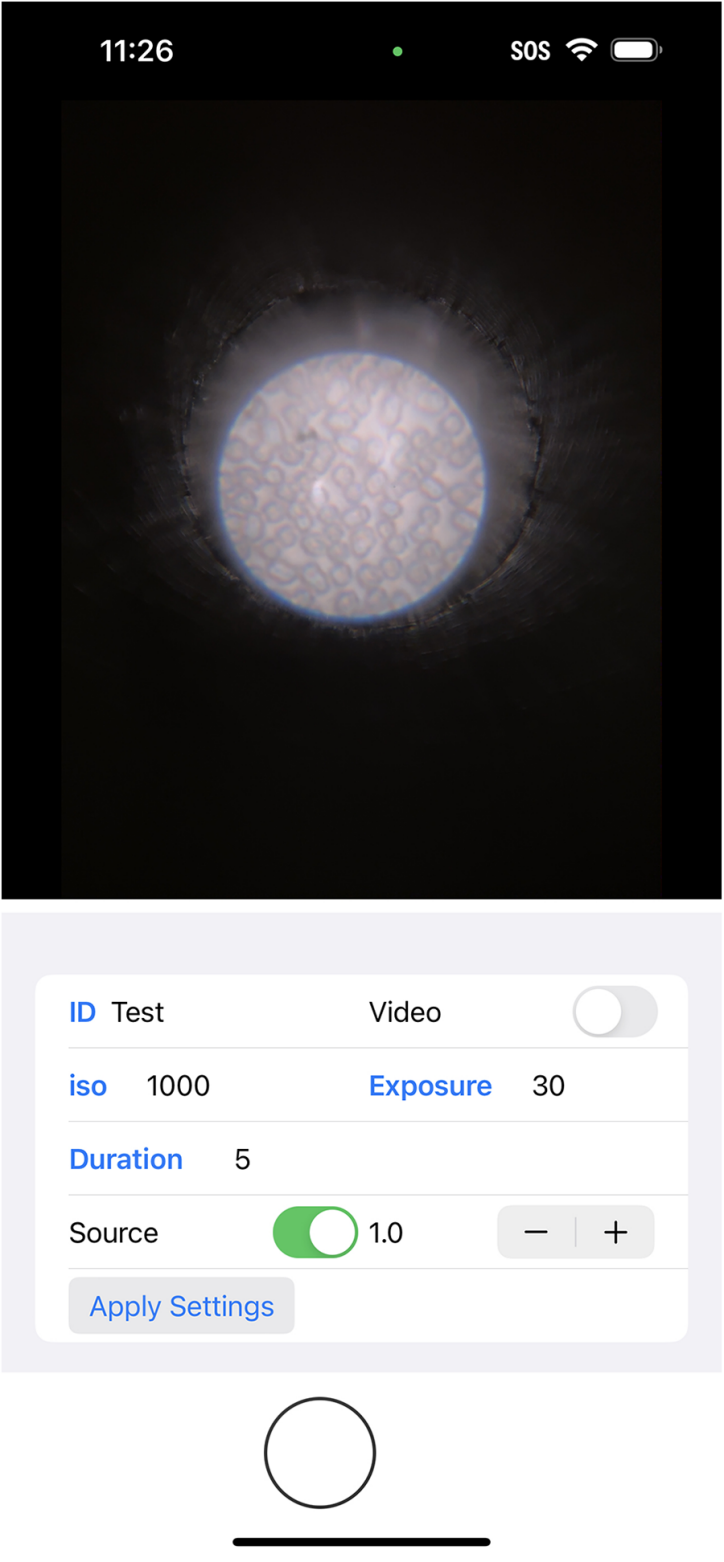
Screenshot of the custom-made iOS application.

During the adaptation process, some adjustments were required to ensure the system’s functionality remained consistent across different platforms. One of the key adjustments involved overcoming the limitation of the iPhone’s built-in camera application, which lacks the ability to keep the flashlight on continuously during photo preparation—an essential feature for locating specific regions of the biosamples that require analysis. Thus, we developed a custom iOS application that enabled the flashlight to remain on constantly with adjustable brightness levels, as shown in Figure 2. This improvement facilitated image field determination while also ensuring that the camera could adjust its focus on the features before taking the pictures. The app also allows for adjustment of the ISO (International Organization for Standardization) value, which controls the camera’s sensitivity to light, and shutter time, which determines the exposure duration. Adjusting these settings helps optimize image quality based on the phone model and the type of analysis required.

**Figure 3:**
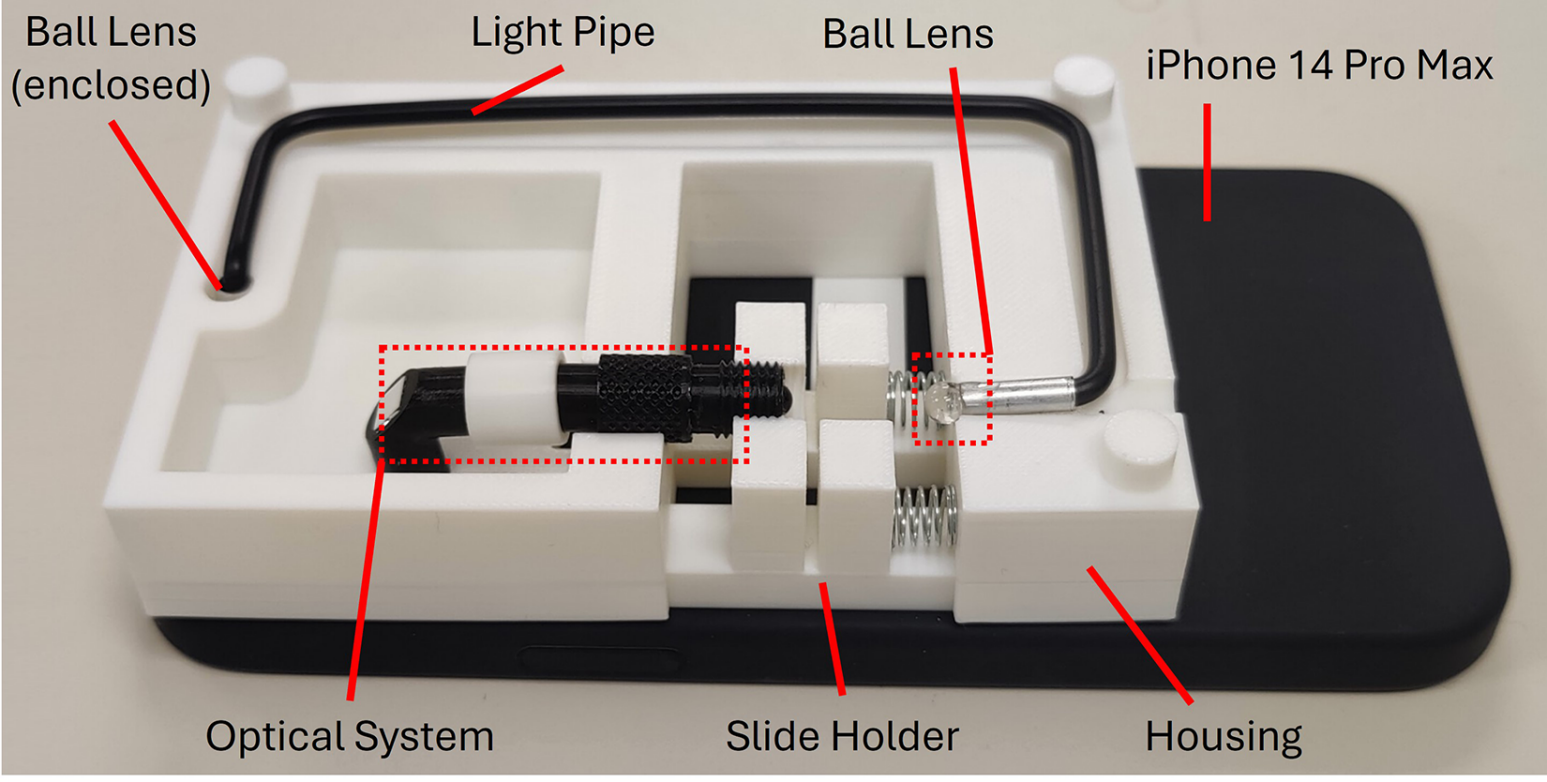
Prototype of μ-phone adapted to an iPhone 14 Pro Max. The ball lens next to the phone flashlight is hidden from view.

However, during the development and testing phase, we encountered limitations with the flashlight’s brightness as well as the light pipe’s efficiency in light transmission. The previous lighting setup was found to be inadequate for providing enough illumination brightness, which resulted in uneven backlight intensity in the image captured. It also resulted in a lot of dirt that built up on the lens system being captured on the images. The accumulation of dirt on various lens components is normal since the optical system is not fully sealed. In most commercial microscopes though, dirt on these surfaces typically does not significantly affect image quality because the bright backlight reduces its visibility. However, in our device, the backlight is not as bright, so any dirt or dust on the lens or optical surfaces is more likely to create noticeable artifacts, such as dimmer areas or visible specks in the image. This issue is particularly pronounced when the dirt is located on the surfaces closest to the sample or light source, where it may even show up as distinct artifacts. Thus, we added two glass beads on both sides of the light pipe. The one on the flashlight end is able to collect more light, while the one on the side is able to focus more light onto the sample, as shown in Figure 3. This brighter illumination significantly enhanced the visibility of cells in the captured images, thereby providing more detailed morphological information about the cells, and improving the accuracy and reliability of the cell counting process.

**Figure 4:**
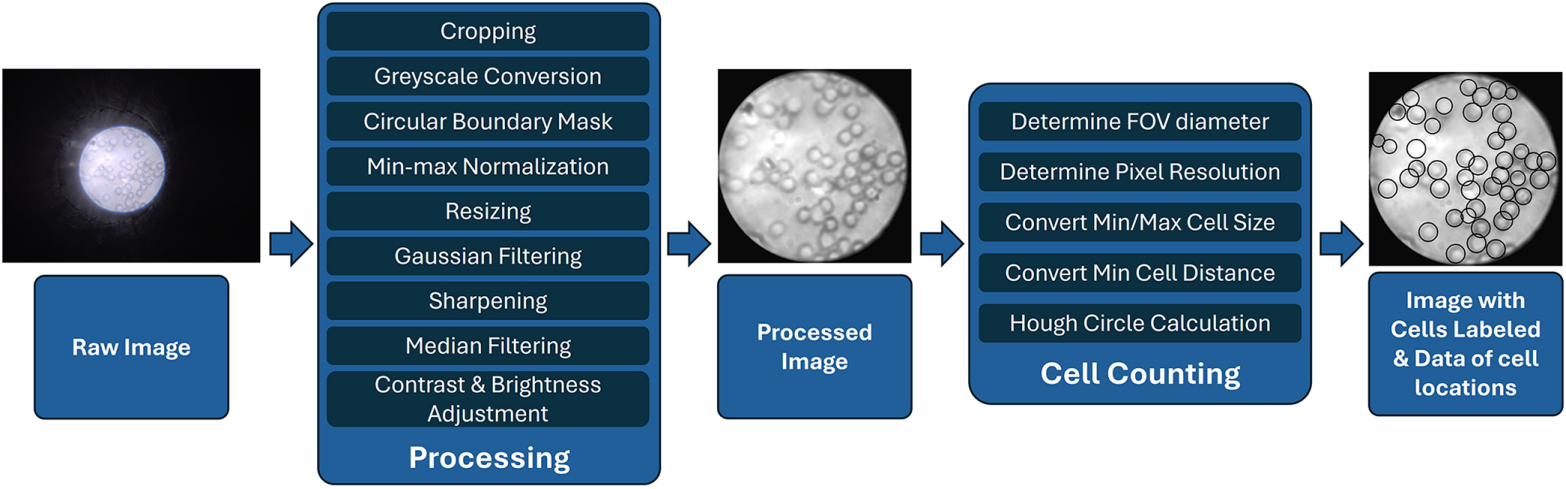
Processing and cell counting flowchart for images taken with μ-phone.

Furthermore, we developed a cell counting algorithm tailored to the images captured using this new system. The algorithm was designed to be simple and efficient, with a streamlined workflow, as shown in Figure 4. The image taken with the custom camera application would be loaded in a Python environment with OpenCV, and would then be cropped to a square shape to exclude all the dark areas. It would then be converted to grayscale before a circular mask would be applied to isolate the field-of-view (FOV) area. The cell boundaries were then enhanced with min-max normalization based on the pixel intensities in the FOV area. The entire picture would then be shrunken to a dimension of 501 by 501 pixels to reduce the calculation workload for all the remaining steps. To achieve optimal image quality for the subsequent cell recognition step, a Gaussian filter with a kernel size of 5×5 was applied to reduce noise, followed by a sharpening filter to enhance the edges of the cells. The sharpening filter used the following 3×3 kernel:

$$\left[\begin{array}{ccc} -1 & -1 & -1\\ -1 & 9 & -1\\ -1 & -1 & -1 \end{array}\right]$$

Next, a median filter with a 5×5 window size was applied to further reduce noise while preserving edges. The image was then processed using Contrast Limited Adaptive Histogram Equalization (CLAHE) with a clip limit of 1.0 and a grid size of 8×8 to enhance local contrast. Finally, the brightness was adjusted by adding a constant bias of 7, and the contrast was fine-tuned with a scaling factor of 1.02, improving the visibility of the cells for accurate detection.

As a preliminary proof-of-concept prototype, a Hough circle algorithm is used to identify targeted cell types. The targeted cell is identified by its minimal and maximal diameter in amount of pixels, as well as the minimum distance between cells. These dimensions can be looked up in units of micrometers and converted to the number of pixels based on the pixel resolution of the image. In our algorithm the minimum radius of 2.5 μm and the maximum radius of 5 μm were used. To reduce the false detection of multiple neighboring circles in addition to the true one, the minimum distance between circles is set to 4.5 μm. The conversion ratio could be calculated using a fixed spacing grid target, as performed in the previous research (16). Similar to the previous research, a complete human blood smear was prepared, and different pictures of the blood smear were collected to validate the cell counting algorithm. All analyzed images were collected from different and non-overlapping areas of the same blood smear slide that was prepared using a drop of finger-pricked blood collected from one of the authors with informed consent. For this project, 22 images were taken as the dataset.

## 3 Results

**Figure 5:**
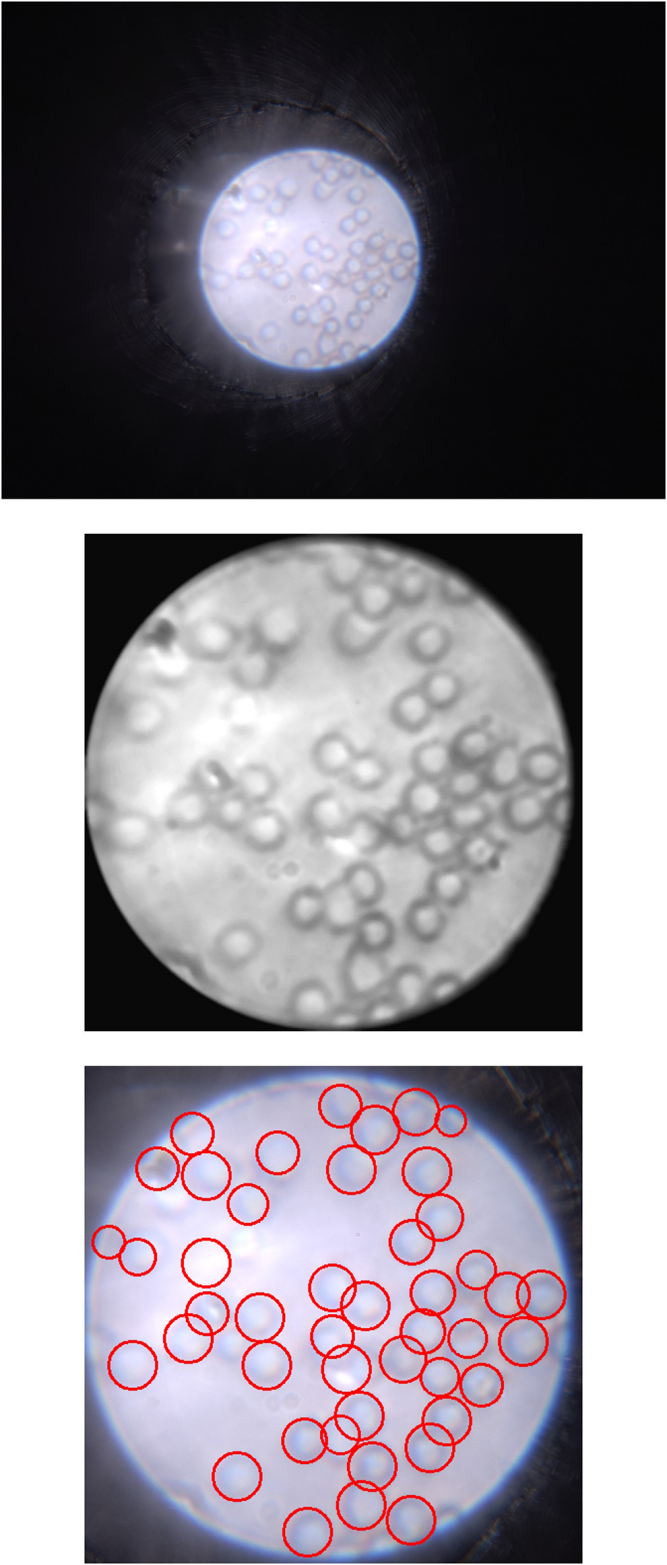
Left: Raw image captured by μ-phone with iPhone 14 Pro Max; Center: Processed image showing more obvious cell edges and shapes; Right: Identified cells with the cell counting algorithm.

An example processing result of a red blood cell image captured using the μ-phone is shown in Figure 5. Since the optical system setup is the same as the previous work, the images taken also have the same pixel resolution and spatial resolution, namely 26.3 px/μm and 2.19 μm, respectively. It can be seen from this image that all the cells can be observed in the raw image, but the image processing enhances the contrast of the edges of the cells, making it possible to classify each cell based on morphological features such as shape, roundness, and diameter. For red blood cells, these features, particularly their circular shape and diameter, are key in distinguishing them and enabling further classification. It also shows that a relatively straightforward algorithm is able to successfully identify and highlight almost all cells in the image.

**Figure 6:**
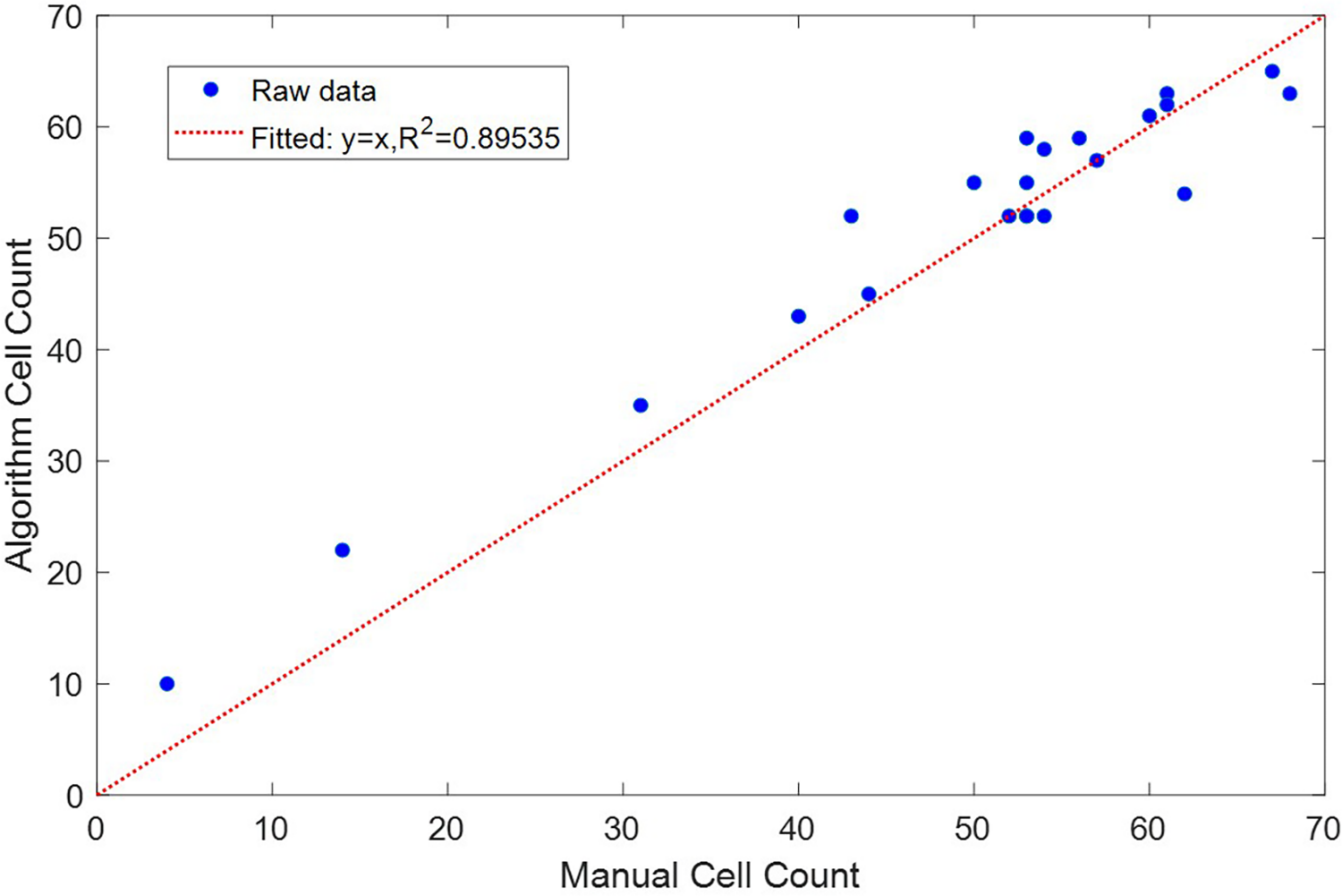
Comparison between cells counted manually and generated by the cell counting algorithm.

To validate the accuracy and consistency of the algorithm, we compared the cell counts generated by the algorithm with the actual cell counts obtained through manual counting for the 22 different images in the dataset. The results showed a linear correlation ($R^{2}=0.89535$) between the actual and algorithm-generated cell counts, as shown in Figure 6. Detailed error analysis data can be found in Table 1. The algorithm achieved a recall of 0.9312 and a precision of 0.8663. The mean absolute error (MAE) was calculated to be 3.36, and the mean squared error (MSE) was 18.27.

**Table 1:** Error analysis of the cell counting algorithm.

Precision (mean ± std)	Recall (mean ± std)	Mean absolute error	Mean squared error
0.8663±0.1265	0.9312±0.0525	3.3636	18.2727

## 4 Discussion

The system demonstrates its potential to perform red blood count and its possibility to perform cell type identification and classification based on morphological features, indicating its capability to revolutionize preoperative testing and address the various challenges associated with current preoperative lab tests.

The R-squared value of 0.89535 indicates a strong linear correlation between the actual cell counts and those generated by the algorithm. The high recall of 0.9312 indicates that the algorithm is able to recognize almost all of the red blood cells. Its relatively lower precision of 0.8663 indicates that it is susceptible to falsely recognizing other artifacts, for example speckles of dirt, as cells. The algorithm also produces a mean absolute error of 3.36 and a mean squared error of 18.27, which suggests that while most predictions are accurate, there are occasional larger discrepancies that could be further optimized.

One of the primary advantages of the system is the simplicity and speed of data collection. The entire algorithm is very lightweight and takes only about 1 s to run, making the procedure extremely fast and accessible. This rapid processing time is beneficial for the widespread adoption of such technologies since it would not impose any extra inconveniences in either technology requirement or time.

The device also shows promise in identifying and classifying different cell types, which is essential for comprehensive preoperative testing. However, to fully realize its potential, a larger database of cell images and classifications is needed. With an expanded database, the accuracy and reliability of the device in distinguishing various cell types can be further improved, making it a more robust and powerful tool for preoperative assessments.

Furthermore, the system also closely mirrors the current clinical standard’s cell-counting process using hemocytometers, which involves counting cells in a defined area with fixed fluid depth and grid spacing. Our system has the potential to automate this process, reducing the need for trained personnel to perform manual counts. However, further hardware and software adaptations, such as compatibility with the hemocytometer chips and automatic calculation of grid spacings, are needed to bring the device closer to clinical standards.

Since this system is an early-stage proof-of-concept prototype, it has a lot of limitations that could be improved in future development. One of the main limitations of the current device is its extremely limited FOV. Enhancing the FOV would allow for the examination of larger sample areas, reducing the need for multiple scans and thus further speeding up the testing process. A wider FOV would also facilitate the detection of rare cell types or anomalies that might be missed in a smaller viewing area. Furthermore, future development direction should also include achieving an adjustable magnification ratio of the device, since the current high magnification ratio is good for visualizing individual cells but lack the ability to visualize larger components like blood clots. Integrating a range of different magnification ratios would certainly expand the capability of the device to more comprehensive usages of other biosamples. The cell counting algorithm would also need further development to achieve better precision and accuracy. The current algorithm is fast but simple, with both Type I and II errors observed, so developing a new algorithm that achieves higher accuracy without sacrificing the processing speed would make the system more reliable.

In addition, future development should also include integrating the camera application with the cell counting algorithm, as it would open up possibilities of analysis for a larger sample size beyond the constraint of an image. Eventually, it might even be possible to annotate live video streams. However, retaining the option to capture images could still be beneficial, especially for providing visual references to physicians and pathologists for additional judgment and reference. Implementing the ability to share images with healthcare providers can also aid in collaborative decision-making and ensure that patients receive the most accurate and comprehensive care.

Another direction for future development and research is to integrate a microfluidic channel or tube, enabling direct observation and analysis of fluid samples without the need for prior sample preparation. The current process of manually preparing blood smears on glass slides presents a challenge, as it requires additional training to ensure proper preparation and handling. In addition, while users can currently draw blood using standard methods, such as finger-pricking and preparing a slide, this approach only allows a fraction of the sample to be observed, with results often extrapolated from this limited view. By incorporating a microfluidic solution, we aim to eliminate the need for manual sample preparation and improve accuracy, as every cell in the fluid sample could be observed and recorded directly.

Future developments could focus on effectively implementing the system into clinical practice. Incorporating training materials directly into the software for healthcare providers and patients could help users quickly become familiar with the system. Additionally, integrating the system’s test results and images into patients’ electronic health records (EHRs) would enable seamless sharing of data with healthcare providers, facilitating faster diagnosis and treatment decisions. These improvements would enhance the practicality of the device and support its integration into preoperative lab testing workflows in both urban and rural environments.

In general, this device has the potential to serve as a promising alternative for integration into preoperative lab test procedures. For care providers, the device simplifies preoperative testing workflows by eliminating the need for extensive laboratory setups, particularly in resource-constrained environments. This reduction in complexity helps medical staff focus on patient care rather than managing laboratory infrastructure. For patients, especially those in rural or remote areas, the system offers an opportunity for frequent, real-time health monitoring without the burden of traveling to distant healthcare facilities. This portability ensures that preoperative testing is not only faster but also more accessible and affordable.

The key contribution of this work lies in the integration of the microscope hardware with custom image analysis software to create a fully functional system. While previous work focused on the hardware, this study presents a deployable solution that automates diagnostic procedures through the custom app and cell counting algorithm. This complete system is designed for real-world use, eliminating the need for specialized personnel and lab infrastructure. By offering a comprehensive, easy-to-use platform, the device significantly reduces the financial and logistical burdens associated with clinical diagnostics. The system’s ability to process data in real time and perform automated analysis makes it a practical alternative to traditional lab tests, particularly in remote and underserved areas.

## Data Availability

The datasets presented in this study can be found in online repositories. The names of the repository/repositories and accession number(s) can be found below: https://github.com/ksong13/muPhone.

## References

[1] Apfelbaum JL Connis RT Nickinovich DG Pasternak LR Arens JF Caplan RA, et al. Practice advisory for preanesthesia evaluation: an updated report by the American Society of Anesthesiologists Task Force on Preanesthesia Evaluation. Anesthesiology. (2012) 116:522–38. 10.1097/ALN.0b013e31823c1067 22273990

[2] Bolenz C Schröppel B Eisenhardt A Schmitz-Dräger BJ Grimm MO. The investigation of hematuria. Dtsch Ärztebl Int. (2018) 115:801. 10.3238/arztebl.2018.0801 30642428 PMC6365675

[3] Johnson R Mortimer A. Routine pre-operative blood testing: is it necessary? Anaesthesia. (2002) 57:914–7. 10.1046/j.1365-2044.2002.02750.x 12190758

[4] Kumar A Srivastava U. Role of routine laboratory investigations in preoperative evaluation. J Anaesthesiol Clin Pharmacol. (2011) 27:174. 10.4103/0970-9185.81824 21772675 PMC3127294

[5] Feely MA Collins CS Daniels PR Kebede EB Jatoi A Mauck KF, et al. Preoperative testing before noncardiac surgery: guidelines and recommendations. Am Fam Physician. (2013) 87:414–8. 23547574

[6] Sherrer M Simmons JW Dobyns JB. Preoperative risk stratification: identifying modifiable risks for optimization. Curr Anesthesiol Rep. (2022) 12:10–25. 10.1007/s40140-022-00519-z

[7] Drancourt M Michel-Lepage A Boyer S Raoult D. The point-of-care laboratory in clinical microbiology. Clin Microbiol Rev. (2016) 29:429–47. 10.1128/CMR.00090-15 27029593 PMC4861988

[8] Wang EY Zafar JE Lawrence CM Gavin LF Mishra S Boateng A, et al. Environmental emissions reduction of a preoperative evaluation center utilizing telehealth screening and standardized preoperative testing guidelines. Resour Conserv Recycl. (2021) 171:105652. 10.1016/j.resconrec.2021.105652

[9] Welke KF Pasquali SK Lin P Backer CL Overman DM Romano JC, et al. Hospital distribution and patient travel patterns for congenital cardiac surgery in the united states. Ann Thorac Surg. (2019) 107:574–81. 10.1016/j.athoracsur.2018.07.047 30248321

[10] Ferschl MB Tung A Sweitzer B Huo D Glick DB. Preoperative clinic visits reduce operating room cancellations and delays. J Am Soc Anesthesiol. (2005) 103:855–9. 10.1097/00000542-200510000-00025 16192779

[11] Wilson ML Fleming KA Kuti MA Looi LM Lago N Ru K. Access to pathology and laboratory medicine services: a crucial gap. The Lancet. (2018) 391:1927–38. 10.1016/S0140-6736(18)30458-6 29550029

[12] Liu J Geng Z Fan Z Liu J Chen H. Point-of-care testing based on smartphone: the current state-of-the-art (2017–2018). Biosens Bioelectron. (2019) 132:17–37. 10.1016/j.bios.2019.01.068 30851493

[13] Okobi E Adigun AO Ozobokeme O Emmanuel O Akinsanya PA Okunromade O, et al. Examining disparities in ownership and use of digital health technology between rural and urban adults in the US: an analysis of the 2019 health information national trends survey. Cureus. (2023) 15(5):e38417. 10.7759/cureus.38417 37273368 PMC10233341

[14] Simpao AF Lingappan AM Ahumada LM Rehman MA Gálvez JA. Perioperative smartphone apps and devices for patient-centered care. J Med Syst. (2015) 39:102. 10.1007/s10916-015-0310-7 26265239

[15] Fontelo P Liu F Yagi Y. Evaluation of a smartphone for telepathology: lessons learned. J Pathol Inform. (2015) 6:35. 10.4103/2153-3539.158912 26167379 PMC4485193

[16] Song K Wu S Peng R Cobb J Adams AT. μ-phone: accessible microscope attachment for smartphones. In: IEEE-EMBS International Conference on Body Sensor Networks (In press) (2024)

